# Distinct anti-proliferative effects of herbal melanin on human acute monocytic leukemia THP-1 cells and embryonic kidney HEK293 cells

**DOI:** 10.1186/s12906-020-02944-1

**Published:** 2020-05-24

**Authors:** Adila El-Obeid, Hala Alajmi, Mashael Harbi, Wesam Bin Yahya, Hamad Al-Eidi, Monira Alaujan, Adil Haseeb, Thadeo Trivilegio, Alshaimaa Alhallaj, Saleh Alghamdi, Abdul-Wali Ajlouni, Sabine Matou-Nasri

**Affiliations:** 1 Cell and Gene Therapy Group, Medical Genomics Research Department, King Abdullah International Medical Research Center, Ministry of National Guard Health Affairs, P.O. Box 22490, Riyadh, 11426 Saudi Arabia; 2 Department of Biobank, King Abdullah International Medical Research Center, Ministry of National Guard Health Affairs, Riyadh, Saudi Arabia; 3 School of Pharmacy, Ahfad University for Women, Khartoum, Sudan; 4 King Saud bin Abdulaziz University for Health Sciences, Riyadh, Saudi Arabia; 5 Toxicology Department, Naif Arab University for Security Sciences, Riyadh, Saudi Arabia; 6 Attosecond-Laser Laboratory, Faculty of Science, Kind Saud University, Riyadh, Saudi Arabia; 7 Core Facility, King Abdullah International Medical Research Center, Ministry of National Guard Health Affairs, Riyadh, Saudi Arabia

**Keywords:** Herbal melanin, THP-1, Human embryonic kidney, HEK293, Toll-like receptor 4, Apoptosis, Cell cycle

## Abstract

**Background:**

Herbal melanin (HM) is a dark pigment extracted from the seed coat of *Nigella sativa L.* and known to exert biological effects via toll-like receptor 4 (TLR4). Recently, TLR4 was described as involved in natural programmed cell death (apoptosis). Tumor and embryonic cells are used as in vitro cellular models for drug and anti-cancer agent screening. To date, no cytotoxic studies have been reported of HM in TLR4-positive acute monocytic leukemia THP-1 cells compared to TLR4-negative human embryonic kidney HEK293 cells.

**Methods:**

We studied the anti-proliferative effects of several HM concentrations on THP-1 and HEK293 cells by evaluating cell viability using the CellTiter-Glo® luminescent assay, assessing the TLR4 expression level, determining the apoptotic status, and analyzing the cell cycle distribution using flow cytometry. Apoptotic pathways were investigated using mitochondrial transition pore opening, caspase activity assays and immunoblot technology.

**Results:**

Low HM concentrations did not affect THP-1 cell viability, but high HM concentrations (62.5–500 μg/mL) did decrease THP-1 cell viability and induced G_0_/G_1_ phase cell cycle arrest. Only at the highest concentration (500 μg/mL), HM slightly increased the TLR4 expression on the THP-1 cell surface, concomitantly upregulated TLR4 whole protein and gene expression, and induced apoptosis in THP-1 cells via activation of the extrinsic and intrinsic pathways. No change of apoptotic status was noticed in TLR4-negative HEK293 cells, although HM decreased HEK293 cell viability and induced cell growth arrest in the G_2_ phase.

**Conclusion:**

HM exerts distinct anti-proliferative effects on human acute monocytic leukemia and embryonic kidney cells mainly through cell cycle interference in a TLR4-independent manner and through apoptosis induction in a TLR4-dependent manner, as observed in only the THP-1 cells.

## Background

Melanins are a family of dark-brown to black, heterogeneous and multifunctional oligomeric pigments found in animals, plants and microorganisms. They cause tissue darkness and protect against solar radiation, including ultraviolet rays, visible light, heat and other types of radiation [1–3]. Herbal melanin (HM) has been extracted from *Nigella sativa L*., an herbaceous plant that is traditionally used in the Middle East and Southeast Asia for the treatment of various diseases including digestive, respiratory, cardiovascular and immune system disorders [4]. HM has been shown to possess various biochemical properties, including antioxidant activity and free radical-scavenging capacity [5]. In addition, the presence of melanin has been associated with various immune responses in animals, plants and invertebrates [6–8]. The biochemical properties of HM align with the use of *Nigella sativa L*. for the treatment of several types of diseases, and reveal potent anti-inflammatory, anti-ulcer and anti-diabetic activities [9–11]. Using a human acute monocytic leukemia cell line THP-1, HM has been demonstrated to exert immuno-modulatory properties through the secretion of interleukin 6 (IL6), and of tumor necrosis factor alpha (TNFα), both anti-tumoral cytokines that contribute to tumor regression, following the activation of transmembrane toll-like receptor 4 (TLR4) [12–15].

The TLRs constitute a group of type I integral membrane receptors that act in the first line of defense in the human innate immune system. They are involved in the clearance of invading pathogens through a protective immune response, or the active programmed cell death (apoptosis) response in eukaryotic cells [16, 17]. Among the 13 members of the TLR family, TLR4 is a cell surface sensor that recognizes and responds to pathogen-associated molecular patterns including lipopolysaccharides (LPS) [17, 18]. TLR4 has been reported to overexpress in inflammatory sites and various cancer tissues through LPS exposure and the following NF-κB and MAPK pathway activation [19–21]. The activation of TLR4 triggers the stimulation of intracellular signaling pathways via adaptor molecules such as MyD88, followed by activation of inflammatory (TLR4/NOD1/p38 MAPK) and cell death signaling pathways [16, 17]. With regards to the cell death-related signaling pathways, TLR4 activation has been reported to lead to apoptosis in neuronal cells, in various types of cancer cells including lung cancer and THP-1 cells through reactive oxygen species (ROS) production, as well as activation of both extrinsic (including mainly caspase-8 cleavage) and intrinsic apoptotic pathways (mitochondrial-dependent pathway resulting in caspase-9 cleavage) [17, 22–24]. Other hallmarks of TLR4-mediated apoptosis were also observed, such as plasma membrane reversion on exposure to phosphatidylserine on the cell surface, and the loss of the plasma membrane integrity [17, 25]. Hence, it has been demonstrated that TLR4 is required for the cytotoxicity of natural products, and also for the toxicity of cytokine-induced killer cells [26]. Recently, it was described that TLR4 is required for the induction of antitumor immune responses, and tumor regression in patients with carcinoma [27]. In addition, TLR4-negative cells such as human embryonic kidney HEK293 cells have been presented as an in vitro cellular model for the investigation of biological effects mediated through TLR4 receptor in comparison with TLR4-positive cells [15], and for drug and anti-cancer agent screening [28].

Recently, high concentrations of aqueous extracts of HM have been reported to exert cytotoxic effects in human cancer cells, such as gastric adenocarcinoma cells, and epithelial cells derived from larynx carcinoma [29, 30]. However, cytotoxic studies of several high concentrations of HM on the growth of human acute monocytic leukemia THP-1 and embryonic kidney HEK293 cell lines have not yet been conducted. Therefore, we investigated the potential cytotoxic and anti-proliferative effects of HM in THP-1 and HEK293 cells.

## Methods

### Chemical compounds

All reagents were obtained from Sigma-Aldrich unless otherwise mentioned.

### Herbal melanin (HM) preparation and chemical analysis

HM was extracted from the *Nigella sativa L.* seed coat and its structure and physicochemical properties were verified using electron spin resonance, Fourier transform infrared, ultraviolet-visible, nuclear magnetic resonance, X-ray diffraction, and X-ray fluorescence experimental techniques [14]. Elemental analysis for the content of carbon, hydrogen and nitrogen in the HM extract confirmed the close similarity of the general characteristics of this extract to eu-melanins as previously described [14, 31]. The HM working solution was prepared as previously described [14].

### THP-1 and HEK293 cell culture

Human acute monocytic leukemia THP-1 (# TIB-202™) and human embryonic kidney HEK293 (# CRL-1573™) cell lines were obtained from the American Type Culture Collection (ATCC, Rockville, MD, USA). THP-1 cells were cultured in a Roswell Park Memorial Institute (RPMI)-1640 medium, while the HEK293 cells were cultured in Dulbecco’s modified Eagle medium (DMEM). Both culture media were supplemented with 10% heat-inactivated fetal bovine serum (FBS), 2 mM glutamine and antibiotics (100 μg/mL streptomycin and 100 IU/mL penicillin). The cultured cells were maintained at 37 °C in a saturated humid air/5% CO_2_-incubator. The viability of the cells used throughout this study was at least 85%. Cells were treated with or without several concentrations of herbal melanin (HM) between 7.8 μg/mL and 500 μg/mL.

### Cell viability assay

THP-1 and HEK293 cells (5 × 10^3^) were seeded in a 96-well plate (Corning Inc., Corning, NY, USA). Two-fold serial dilutions of the HM extracts were prepared in complete medium to obtain final concentrations ranging from 7.8–500 μg/mL and added to the cells in triplicate. The wells containing only cells with a complete medium were considered as controls. After 24–48 and 72 h of incubation, cell viability was assessed using the CellTiter-Glo® assay kit (Promega Corporation, Madison, WI, USA) according to the manufacturer’s instructions. Briefly, the cell viability assessment was based on the quantification of the amount of ATP present, a molecular indicator of metabolically active cells. The CellTiter-Glo® assay generated a “glow-type” luminescent signal produced by luciferase that was proportional to the percentage of living cells.

### Fluorescence-activated cell sorting (FACS) analysis

The cell cycle distribution was analyzed based on the amount of DNA stained by propidium iodide (PI). Briefly, untreated and treated cells (1 × 10^6^) were washed with PBS and centrifuged at 500×*g* for 5 min, then the cells were fixed with cold 70% ethanol for 1 h. The cells were washed with PBS and centrifuged at 500×*g* for 5 min. A final concentration of 0.2 mg/mL RNase A was added to the cells for 1 h of incubation at 37 °C. A final concentration of 10 μg/mL PI was added to the cells for 15 min in the dark at room temperature. Excess PI was removed by washing the cells twice with PBS and centrifugation. After the second centrifugation, cells were re-suspended in 200 μL PBS and 10,000 cells were analyzed on a Becton Dickinson (BD) FACScanto II flow cytometer. The amount of DNA was evaluated using the BD FACSDiva™ software, and cell cycle distribution was determined using the ModFit LT™ program (version 5.0.9; https://www.vsh.com/products/mflt/downloadMFTrialForm.asp, accessed 18-11-2018).

To assess TLR4 cell surface expression, THP-1 and HEK293 cells (0.5 × 10^6^) were washed with phosphate-buffered saline (PBS) supplemented with 2% FBS and centrifuged at 300×*g* for 10 min. After centrifugation, 10^5^ cells were re-suspended in 20 μL of PBS-2% FBS, then 0.1 μg of mouse anti-TLR4 antibody-fluorescein-isothiocyanate (FITC; #ab45126, Abcam, Cambridge, UK) or IgG_2b_,k-FITC (#ab18427, Abcam) were added and the mixture was kept on ice for 30 min. Excess antibody was removed by washing the cells twice with PBS-2% FBS and centrifugation. After the second centrifugation, cells were re-suspended in 200 μL PBS for FACS analysis. Only viable cells established using forward and side scatter parameters were used for analysis.

Apoptosis was determined using the Becton Dickinson (BD) Annexin V apoptosis detection kit (BD Biosciences, San Jose, CA, USA) according to the manufacturer’s instructions. Briefly, 10^5^ cells were pelleted and re-suspended in 100 μL of 1× binding buffer. Five microliters of Annexin V conjugated to FITC was added to each sample and left at room temperature for 15 min in the dark. The cells were first washed with 2 mL of 1× binding buffer, pelleted and then re-suspended in 200 μL of 1× binding buffer. Five microliters of the DNA dye propidium iodide (PI, viability staining solution) conjugated to phycoerythrin (PE) was added to each sample and the cells were immediately analyzed by flow cytometry. Early and late apoptotic cells were characterized by the binding of Annexin V to phosphatidylserine (considered as Annexin V-positive (^+^) cells), with either no detection (considered as PI-negative (^−^) cells) or detection of DNA (considered as PI^+^-cells), and presenting the cell death phenotypes Annexin V^+^/PI^−^ (early apoptosis) and Annexin V^+^/PI^+^ (late apoptosis), respectively. Viable cells were characterized by Annexin V^−^/PI^−^ status, while necrotic cells were characterized by Annexin V^−^/PI^+^ status.

### Confocal laser scanning microscopy

To check the potential involvement of both extrinsic and intrinsic apoptotic pathways in HM-induced apoptosis in THP-1 cells, caspase-3/7 activity and mitochondrial transition pore assays were performed according to the manufacturer’s instructions using the Image-iT® LIVE Red Caspase-3 and Caspase-7 Detection Kit (Molecular Probes, Eugene, OR, USA), and the Image-iT® LIVE Mitochondrial Transition Pore Assay Kit (Molecular Probes), respectively. Caspase activity was considered positive if cells exhibited a red fluorescence. Mitochondrial transition pore activity was deemed positive if cells displayed a quenching mitochondrial calcium green fluorescence caused by cobalt entering the mitochondria. All fluorescent dyes were visualized using an LSM780 confocal laser scanning microscope (Carl Zeiss Microscopy GmbH, Jena, Germany).

### Preparation of cell lysates and immunoblot analysis

Untreated and HM-treated THP-1 cells (1.5 × 10^6^) were washed with cold PBS. Immediately afterwards, total proteins were extracted after lysing the cells with 80 μL/well of ice-cold NP40 lysis buffer (pH 7.5) containing 1 mM phenylmethylsulfonyl fluoride (PMSF) and a 1% cocktail of protease inhibitors. Cell lysate preparation, protein separation by 12% sodium dodecyl sulfate-polyacrylamide gel electrophoresis (SDS-PAGE) and transfer of separated proteins to polyvinylidene difluoride membranes were performed as previously described [32]. Membranes were stained with the following primary antibodies diluted in a blocking buffer overnight at 4 °C on a rotating shaker: rabbit monoclonal antibody to TLR4 (#ab13867, 1:1000 dilution; provided by Abcam); mouse monoclonal and rabbit polyclonal antibodies to apoptotic proteins such as pro-caspase-8 (which also detects cleaved caspase-8, #8005; 1000 dilution), pro-caspase-9 (#9508, 1: 1000 dilution) and cleaved caspase-9 (#9505, 1:1000 dilution; Cell Signaling); and mouse monoclonal antibodies to GAPDH [6C5] (ab8245, 1:2000 dilution; Abcam). After washing five times for 10 min in TBS-Tween at room temperature, membranes were stained with LI-COR infrared fluorescent IRDye®680 RD goat anti-rabbit and IRDye®800 RD goat anti-mouse secondary antibodies diluted in TBS-Tween containing 3% bovine serum albumin (1:1000 dilution) for 1 h at room temperature with continuous mixing. After a further five washes in TBS-Tween, proteins were visualized using Odyssey CLx scanners (LI-COR Biosciences, Lincoln, NE), and analyzed using Image J software (http://rsbweb.nih.gov/ij/index.html).

### RNA extraction and RT-qPCR

Extraction of total RNA from untreated and treated THP1 cells (3 × 10^6^) was performed using the Illustra RNAspin Mini Isolation Kit (GE Healthcare, Little Chalfont, UK). Total RNA was used to monitor the mRNA expression of human *TLR4*. Complementary DNA (cDNA) was produced from total RNA extracts using the SuperScript first-strand kit (Invitrogen – Life Technologies, Carlsbad, CA, USA) and performed in a Tetrad2 Thermal Cycler (Bio-Rad Laboratories, Hercules, CA, USA). Primer pairs (Invitrogen) and the sequences used were: 5′-GAA GCT GGT GGC TGT GGA-3′ (sense) and 5′-TGA TGT AGA ACC CGC AAG-3′ (antisense) for human *TLR4*, as previously described [33]. The *β-actin* primer sequences used were 5′-TGA TGA CAT CAA GAA GGT GGT GAA G-3′ (sense) and 5′-TCC TTG GAG GCC ATG TGG GCC AT-3′ (antisense). Real-time PCR was done using the QuantiTect Reverse Transcription kit containing PCR SyberGreen Master Mix (Qiagen, Hilden, Germany) to quantitatively monitor the mRNA expression of *TLR4* compared with *β-actin* gene expression (a house-keeping gene used as an internal control). For each analysis, a negative control was prepared using all the reagents except the cDNA template. All the reactions were run in triplicate.

### Statistical analysis

All values were expressed as means ± standard deviation (SD). Statistical differences were estimated using Student’s *t*-Test. Values of *p* < 0.05 were considered significant.

## Results

### HM decreases THP-1 and HEK293 cell viability in a dose-dependent manner

**Fig. 1:**
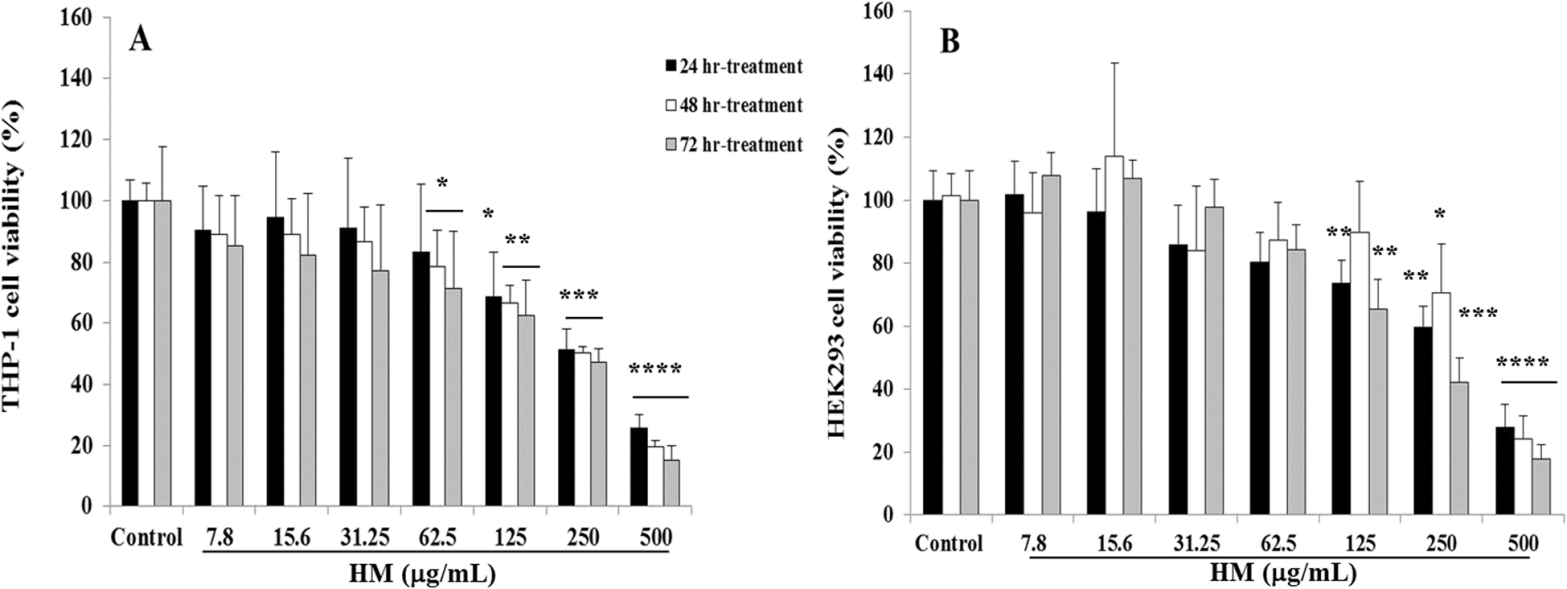
Effects of herbal melanin (HM) on the viability of THP-1 (**a**) and HEK293 (**b**) cells. Cells were treated with 7.8–500 μg/mL of HM for 24–48 and 72 h. Cell viability was determined using the CellTiter-glo® kit. Control comprises untreated cells. Bar graph presents the percentage cell viability, and results are presented as mean ± SD of three independent experiments. (*), (**), (***), and (****) signify a statistically significant difference (*p* < 0.05, *p* < 0.01, *p* < 0.001, and *p* < 0.0001, respectively) compared with the control

To study the cytotoxic effects of HM in cancer and embryonic cells, we tested several high concentrations of HM on the viability of human acute monocytic leukemia THP-1 and embryonic kidney HEK293 cells. Both THP-1 and HEK293 cells were incubated in the presence or absence of different concentrations (7.8–500 μg/mL) of HM for 24, 48 and 72 h of treatment. Cell viability was determined based on the detection of ATP, a molecular indicator of metabolically active cells. Exposure of THP-1 cells to HM at a concentration above 62.5 μg/mL significantly decreased cell viability after 24 h of treatment in a dose- and time-dependent manner (Fig. 1a). At the highest HM concentration (500 μg/mL), a decrease in THP-1 cell viability of 80–90% was noticed at all time-points, compared with the high viability of untreated cells (Fig. 1a). A high percentage of HEK293 cell viability was observed in the presence of HM at concentrations below 125 μg/mL, indicating no cytotoxicity compared with untreated control cells (Fig. 1b). A decrease in the viability of HEK293 cells by 70–80% (*p* < 0.0001) was observed in the presence of high HM concentration (500 μg/mL) after 24 h of treatment, compared with untreated control HEK293 cells (Fig. 1b).

### Herbal melanin (HM) induced THP-1 cell growth arrest in G_0_/G_1_ and HEK293 cell growth arrest in G_2_

**Fig. 2:**
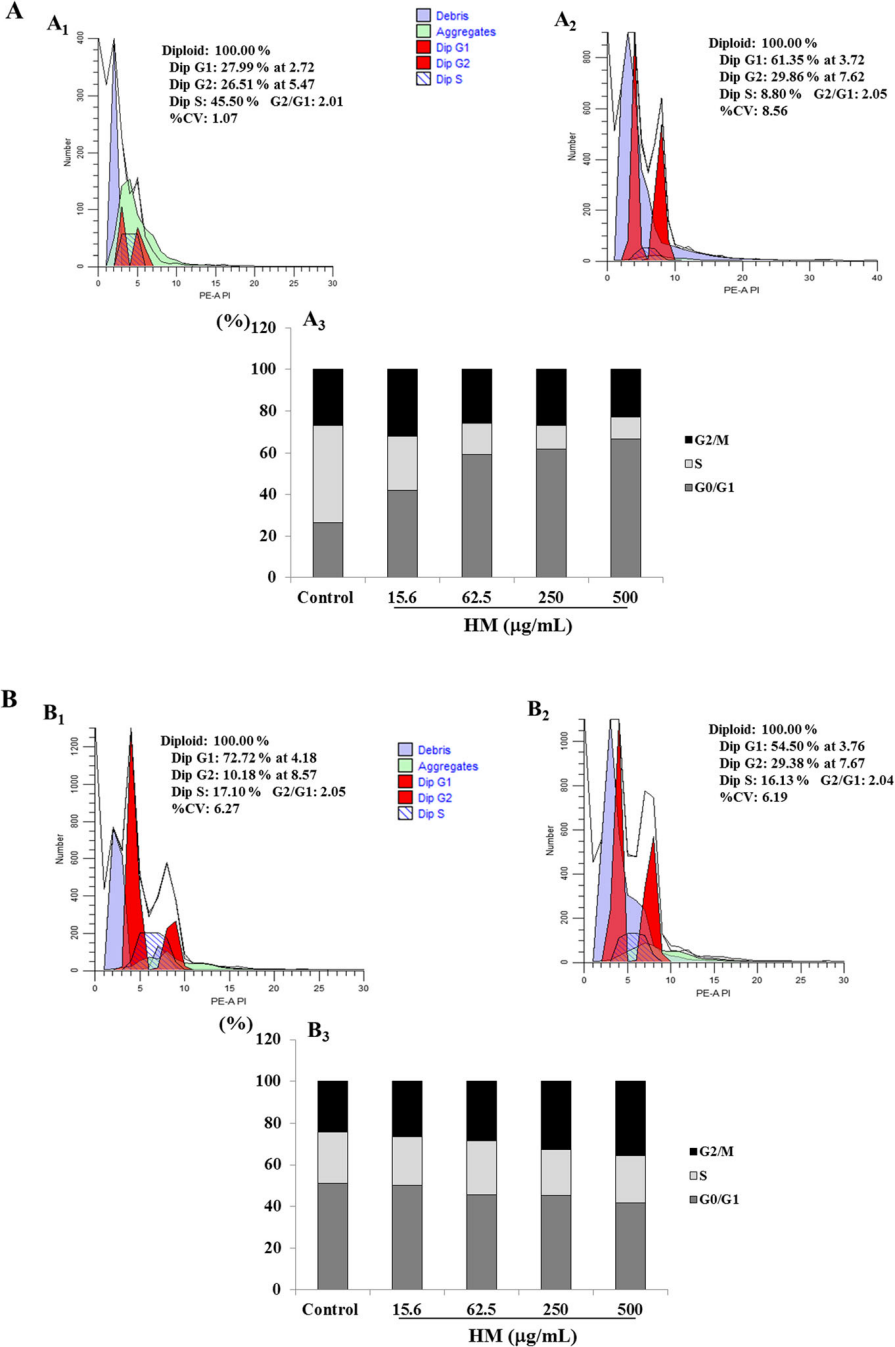
Effects of HM on cell cycle progression in THP-1 (**A**) and HEK293 (**B**) cells. Representative histograms (generated using ModFit LT™ version 5.0.9) of the distribution of cells in the cell cycle, based on DNA staining using propidium iodide (PI) and fluorescence-activated cell sorting (FACS) analysis, after 24 h of incubation of untreated cells (THP-1, A.1; HEK293, B.1) or cells treated with 500 μg/mL HM (THP-1, A.2; HEK293, B.2). Bar graphs present the percentage of cells at each stage of the cell cycle after 24 h of incubation of THP-1 (A.3) and HEK293 (B.3) cells in the presence or absence of various concentrations of HM. Results are presented as means based on three independent experiments

To investigate one of the possible mechanisms of cell death underlying the cytotoxic effects of HM in both THP-1 and HEK293 cells observed at various HM concentrations, the cell cycle was analyzed after staining DNA with PI. The amount of DNA detected in each cell allows the percentage of cells at each phase of the cell cycle to be determined and analyzed using flow cytometry. After 24 h of THP-1 cell treatment with various concentrations of HM, a concomitant increase of the percentage of cells at G_0_/G_1_ phase was observed in THP-1 cells treated with HM at concentrations of 62.5 μg/mL and above, compared with the percentage of cells detected in untreated THP-1 cells (Fig. 2A). In the same conditions, a significant decrease in the percentage of cells in S phase was observed in the THP-1 cells treated with HM concentrations above 62.5 μg/mL (Fig. 2A). No change in the percentage of THP-1 cells in G_2_ phase was observed under any of the tested conditions (Fig. 2A). With regards to HEK293 cells exposed to various HM concentrations and incubated for 24 h, an increase in the percentage of HEK293 cells in G_2_ was observed, accompanied by a decrease in the percentage of cells in G_1_ (Fig. 2B). There was no change in the percentage of HM-treated HEK293 cells in S phase (Fig. 2B).

### High HM concentrations up-regulate TLR4 protein expression in THP-1 cells

**Fig. 3:**
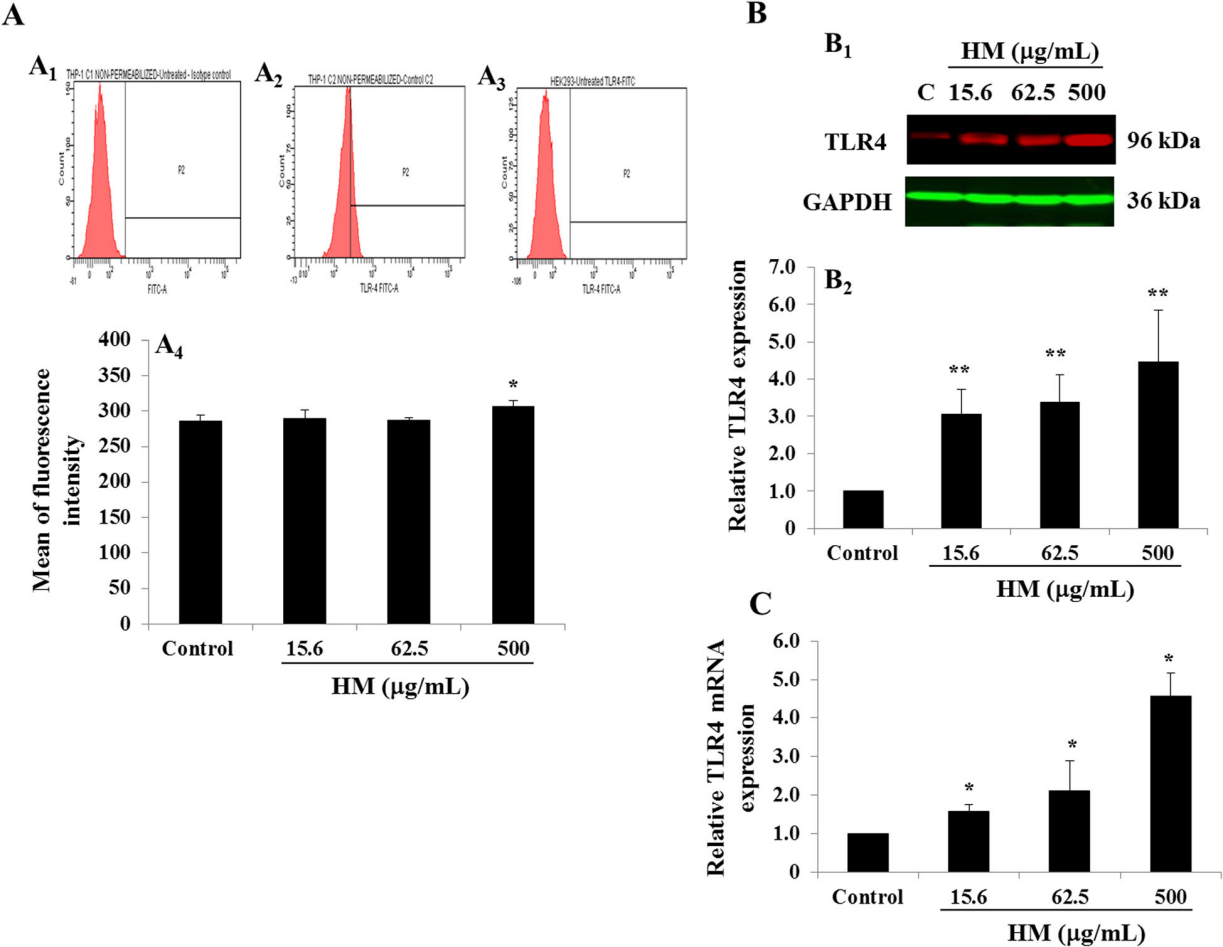
Effect of HM on TLR4 expression in THP-1 cells. **a** Representative histograms showing isotype control antibody FITC-conjugated IgG_2b_ (A.1) used to establish settings for the fluorescence-activated cell sorting (FACS) instrument, untreated TLR4-positive THP-1 cells (A.2), and untreated TLR4-negative HEK293 cells (A.3). Bar graph (A.4) shows the percentage of TLR4-positive cells (determined by FACS analysis) in HM-treated THP-1 cells, compared with untreated cells (control). **b** Representative immunoblot (B.1) showing the effect of various concentrations (15.6, 62.5, and 500 μg/mL) of HM on TLR4 protein expression in THP-1 cells after 24 h of incubation, compared with untreated cells (control). Bar graph (B.2) shows the relative protein expression levels of TLR4, calculated as a ratio of GAPDH expression (the loading control). **c** Bar graph shows the relative expression levels of TLR4 mRNA, calculated as a ratio of the expression of the house-keeping gene *β-actin*. Results are presented as the mean ± SD of three independent experiments. (*) and (**) signify a statistically significant difference (*p* < 0.05 and *p* < 0.01) compared with the control

The TLR4 receptor, belonging to the TLR family of proteins, known to be important in the immune system, has recently been demonstrated to be a site of apoptosis induction [17]. To check whether HM modulates the expression of the TLR4 receptor, FACS analysis, immunoblotting and RT-qPCR were applied after 24 h of THP-1 cell treatment. FACS analysis was performed to evaluate TLR4 expression on the surface of the THP-1 cells in the presence of various concentrations (7.8–500 μg/mL) of HM. Compared with the isotype control IgG_2b_,k-FITC-stained cells (Fig. 3A.1), TLR4 was weakly detected on the surface of untreated THP-1 cells (Fig. 3A.2). As expected, no TLR4 was detected on the surface of HEK293 cells (Fig. 3A.3). At most concentrations used, HM had no effect on TLR4 receptor expression on the cell surface, however, a significant (*p* = 0.03) increase was observed in 500 μg/mL HM-treated THP-1 cells compared with the basal TLR4 expression level detected on the surface of untreated control THP-1 cells after 24 h (Fig. 3A.4). Considering the whole protein extract, increasing concentrations of HM (15.6, 62.5, and 500 μg/mL) upregulated TLR4 protein expression in THP-1 cells in a dose-dependent manner, compared with the basal TLR4 expression level detected in untreated THP-1 cells (Fig. 3B). This up-regulation of TLR4 expression by various concentrations of HM was confirmed at the RNA level using RT-qPCR (Fig. 3C).

### High HM concentrations induce apoptosis in TLR4-positive THP-1 cells and not in TLR4-negative HEK293 cells

**Fig. 4:**
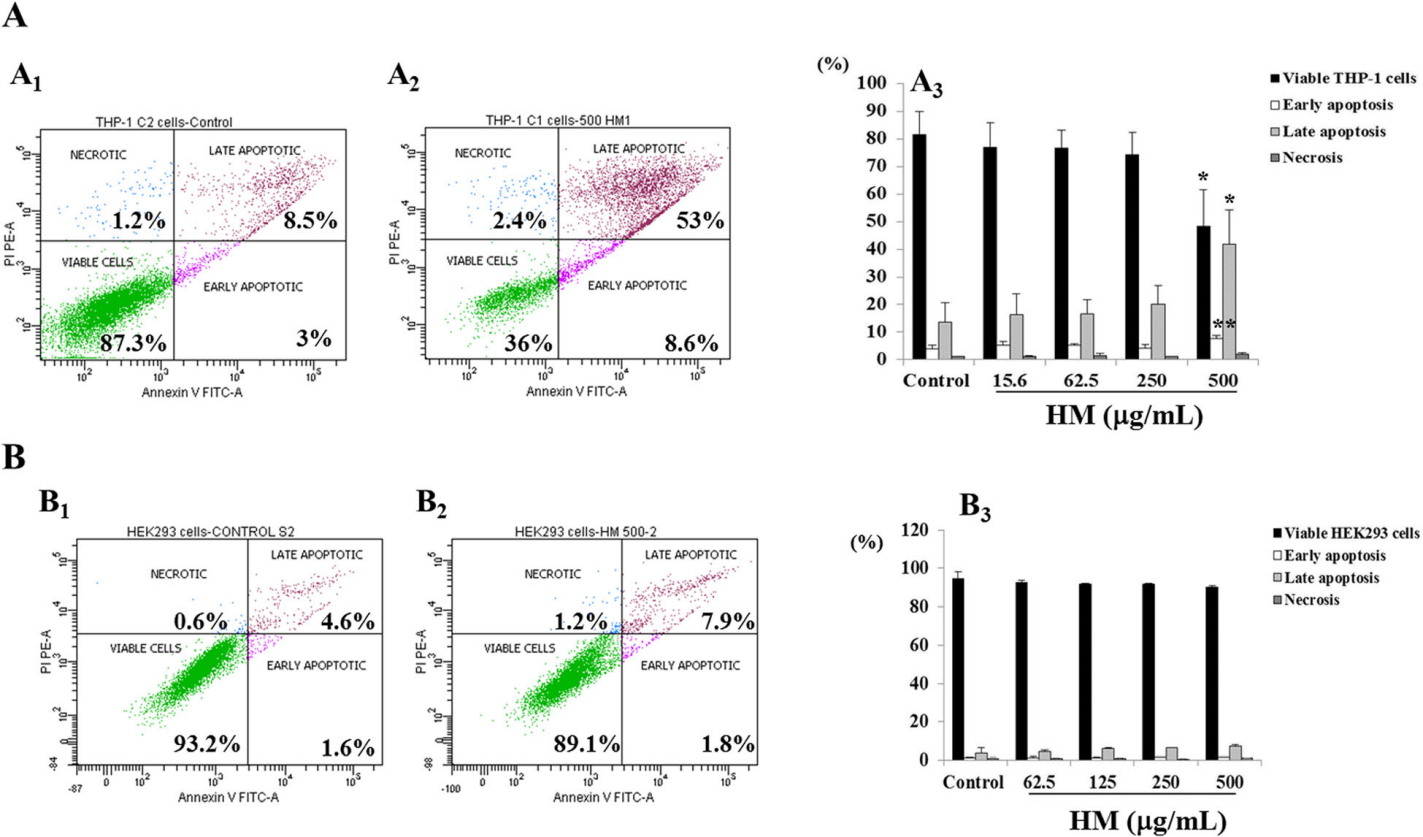
Effects of HM on the induction of apoptosis in THP-1 cells. Cells were treated with 7.8–500 μg/mL of HM for 24 h followed by apoptosis detection using fluorescence-activated cell sorting (FACS) analysis, based on double-staining with Annexin V-fluorescein isothiocyanate (V)/propidium iodide (PI)-phycoerythrin. Representative dot plots showing the percentage of viable cells (lower left, Annexin V^−^/PI^−^), cells in early apoptosis (lower right, Annexin V^+^/PI^−^), cells in late apoptosis (upper right, Annexin V^+^/PI^+^), and necrosis (upper left, Annexin V^−^/PI^+^), compared with untreated control THP-1 cells (A.1), THP-1 cells treated with 500 μg/mL of HM (A.2), untreated HEK293 cells (B.1), and HEK293 cells treated with 500 μg/mL of HM (B.2). Bar graphs present the percentage of viable, early apoptotic, late apoptotic and necrotic cells of THP-1 (A.3) and HEK293 cells (B.3) in the presence or absence of (15.6, 62.5, and 500 μg/mL) HM after 24 h of treatment, based on three independent experiments. (*) and (**) signify a statistically significant difference (*p* < 0.05 and *p* < 0.01) compared with the control

As another potential cell death mechanism underlying the anti-proliferative effect of HM in THP-1 cells, we determined the apoptotic status of these cells based on the binding of fluorescently labeled Annexin-V to externalized plasma membrane phosphatidylserine, after 24 h of cell treatment with various concentrations of HM. THP-1 cell exposure to HM at a concentration below 500 μg/mL did not modify the apoptotic status of the treated cells compared with healthy untreated control cells (Fig. 4). After 24 h of THP-1 cell treatment with the highest concentration (500 μg/mL) of HM, we also observed that 7.8% (*p* = 0.007) of the THP-1 cell population was in early apoptosis, with 41.8% (*p* = 0.01) of the cell population in late apoptosis, compared with untreated control cells (Fig. 4A). A negligible percentage of necrotic cells was detected in all conditions (Fig. 4A). In order to highlight the importance of TLR4 in HM-induced apoptosis in THP-1 cells, TLR4-negative HEK293 cells were treated with various concentrations (62.5, 125, 250, and 500 μg/mL) of HM for 24 h of incubation, then analyzed using FACS. No induction of apoptosis was observed, even after exposing HEK293 cells to the highest concentration of HM (Fig. 4B).

**Fig. 5:**
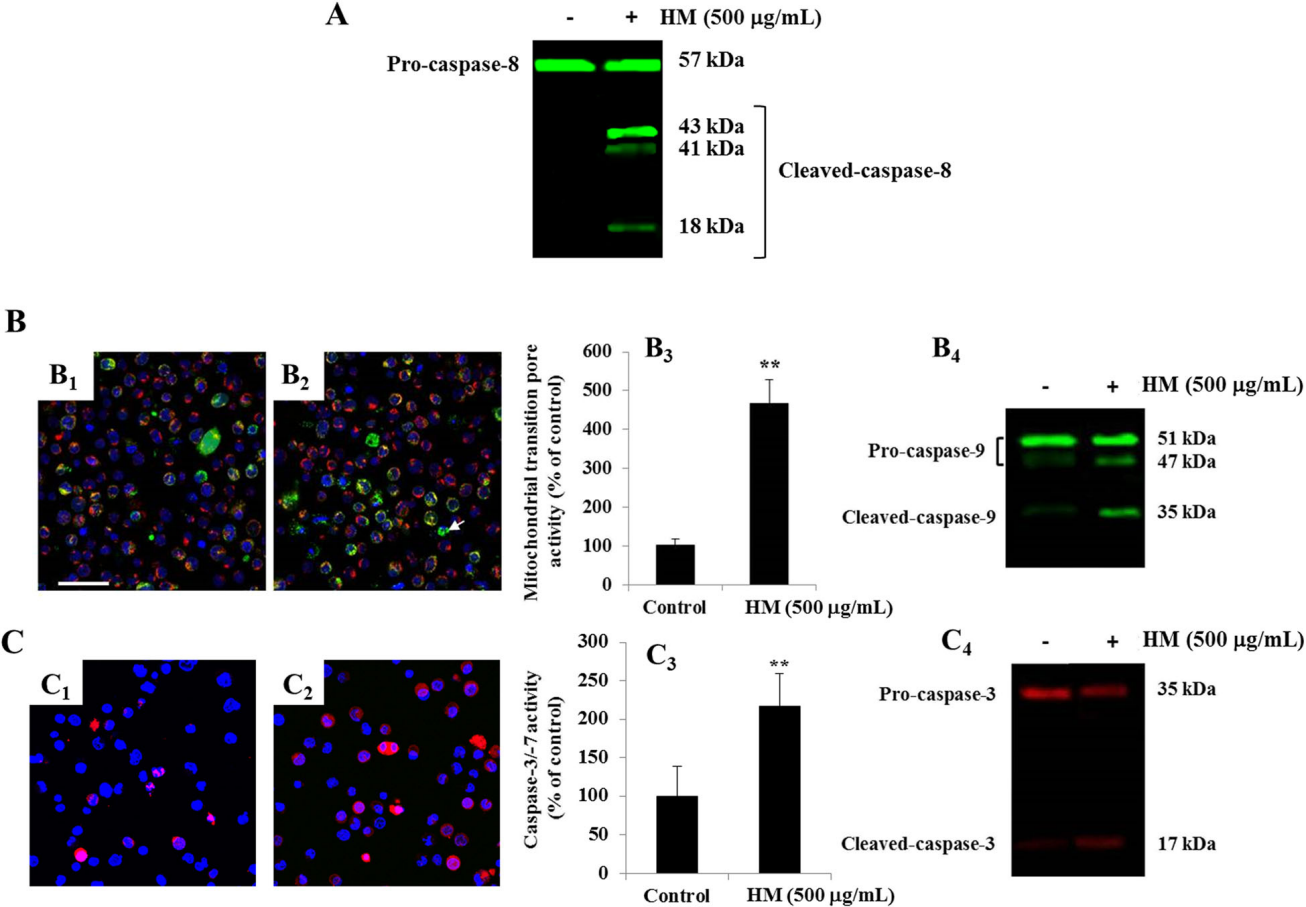
Involvement of extrinsic and intrinsic pathways in HM-induced apoptosis in THP-1 cells. **a** Representative immunoblot showing the detection of pro-caspase-8 expression in untreated THP-1 cells, and the detection of cleaved caspase-8 fragments – hallmarks of extrinsic pathway activation in (500 μg/mL) HM-treated THP-1 cells. **b** Representative photomicrographs showing the permeability of the mitochondrial membrane in untreated THP-1 cells (B.1) or cells treated with 500 μg/mL of HM (B.2) after 24 h of incubation. Increased permeability of the mitochondrial membrane is indicated by arrows showing quenched green fluorescence of calcein (B.2) in (500 μg/mL) HM-treated THP-1 cells. Bar graph (B.3) shows the percentage of apoptosis based on green fluorescence-positive cells for mitochondrial transition pore activity. The representative immunoblot (B.4) showing pro- and cleaved-caspase-9 confirms intrinsic apoptotic pathway activation in (500 μg/mL) HM-treated THP-1 cells. **c** Representative photomicrographs showing caspase-3/7 activity indicated by red fluorescence and detected in either untreated THP-1 cells (C.1) or cells treated with 500 μg/mL of HM (C.2) after 24 h of incubation. Bar graph (C.3) shows the percentage of apoptotic cells based on red fluorescence-positive cells for caspase-3/7 activity. Representative immunoblot (C.4) showing pro and cleaved caspase-3 (B.4) detected in THP-1 cells after 24 h of treatment, with or without 500 μg/mL of HM. (**) signifies a statistically significant difference (*p* < 0.01) compared with the control after 24 h of incubation. Scale bar = 50 μm

To further investigate HM-induced apoptosis in THP-1 cells, we assessed pro- and cleaved-caspase-8/− 9 expression, conducted a mitochondrial transition pore assay, and determined caspase-3/7 activity to explore both extrinsic and intrinsic apoptotic pathways. Using immunoblotting, the cleavage of caspase-8 into three cleaved-caspase-8 fragments was detected in HM-treated THP-1 cell lysate, compared with the concomitant expression of pro-caspase-8 observed in only untreated THP-1 cells (Fig. 5A). Activation of the intrinsic apoptotic pathway was confirmed by the apparition of cleaved-caspase-9 fragments in HM-treated THP-1 cell lysate, compared with the low level of cleaved-caspase-9 fragments detected in only untreated THP-1 cells (Fig. 5B_4_). A significant increase in the mitochondrial transition pore assay activity in HM (500 μg/mL) was observed, compared with the low level of activity determined in healthy untreated cells (Fig. 5B.1–3). A lower activity of executioner caspase-3/7 was detected in HM-treated THP-1 cells, compared with the untreated control cells (Fig. 5B.1–3). Caspase-3 activity was confirmed by the apparition of cleaved-caspase-3 fragments in HM-treated THP-1 cell lysate, compared with the negligible amount of cleaved-caspase-3 detected in untreated THP-1 cells (Fig. 5C.4).

## Discussion

Most conventional neo-adjuvant anti-cancer agents are toxic with a high risk of killing normal cells. This has led to the exploration of the induction of the cell cycle arrest and of natural programmed cell death (i.e. apoptosis) as effective approaches for cancer therapy that avoids triggering inflammation and side effects due to damaged and necrotic cells [34–36]. It is also noteworthy that these conventional anti-cancer agents are expensive and often unaffordable [37]. Consequently, there is an interest in the use of complementary and alternative medicine, such as using herbal extracts as anti-cancer agents, because they are generally free of the deleterious side effects associated with conventional agents, and mostly inexpensive [38]. The screening of anti-cancer agents on embryonic cells presenting cancer-like characteristics such as human embryonic kidney HEK293 cells has been of interest as well [28]. In the current study, we investigated the anti-proliferative effects of various high concentrations of HM on human acute monocytic leukemia THP-1 and embryonic kidney HEK293 cell lines, as observed by the concomitant decrease in the viability of both treated THP-1 and HEK293 cells. It was revealed that HM exerted distinct anti-proliferative effects on both cells including the induction of THP-1 cell growth arrest in G_0_/G_1_ phase and the induction of HEK293 cell growth arrest in G_2_ phase. At the highest concentration of HM (500 μg/mL), apoptosis in THP-1 cells was induced in a TLR4-dependent manner, through the activation of both caspase-dependent extrinsic and mitochondrial-dependent intrinsic pathways.

In the present study, the anti-proliferative effects of HM on b oth THP-1 and HEK293 cells, especially at high HM concentrations resulted in similar cytotoxic potentials in both cells. In concordance with the anti-proliferative effect of HM in THP-1 cells, we previously reported a decrease in the production of the survival protein vascular endothelial growth factor (VEGF) in HM-treated monocytes; this observation can be correlated with the inhibitory effect of HM on THP-1 cell viability [14]. The detection of a down-regulation of the VEGF amount in HM-treated HEK293 cells could confirm the decrease of HEK293 cell viability observed in the presence of high HM concentrations. The anti-proliferative effects of high concentrations (500–1000 μg/mL) of HM were also reported in human carcinoma cells [30]. Previously, studies on the effect of melanin on cell growth were performed using melanin extracts from sources other than *Nigella sativa* L. Contradicting results were reported, demonstrating both positive and negative effects of melanin on osteoblastic MG-63 cells, a skin cancer cell line and normal human dermal cell viability, respectively [39–41]. The decrease in cell viability observed at increasing concentrations (from 62.5 μg/mL) of HM suggested the induction of apoptosis and of cell cycle arrest as potential cell death mechanisms underlying the anti-proliferative effects of HM on both THP-1 and HEK293 cells.

As one of the cell death mechanisms involved in HM-mediated anti-proliferative effects on both THP-1 and HEK293 cells, we observed that HM caused a decrease in cell viability by arresting the progression of the THP-1 cell cycle in G_0_/G_1_ phase and by arresting the progression of HEK293 cell cycle in the G_2_ phase. Previously, the uptake of melanin in epidermal keratinocytes was demonstrated to reduce cell proliferation through G_0_/G_1_ cell cycle arrest associated with a decrease in the expression of cell cycle-dependent proteins, including cyclin-dependent kinase 1 (CDK1), cyclin E, cyclin A, and cyclin B [42]. Down-regulation of these cell cycle-related proteins was observed after epidermal cell treatment with 50–70 and 100 μg/mL of melanin [42]. An assessment of the expression of these cyclin-dependent kinases would confirm HM-induced growth arrest of THP-1 cells in G_0_/G_1_ and of HEK293 cells in G_2_.

Demonstrated as the main receptor for HM [15], the TLR4 expression level has been assessed in the present study on the cell surface using FACS analysis and in whole protein extract using Western blot technology. As expected, HEK293 cells did not express TLR4 while high HM concentrations concomitantly up-regulated expression of the *TLR4* gene and its protein product in THP-1 cells. These latter findings are in agreement with a recent study, which demonstrated that high concentrations of LPS, major TLR4 agonists, up-regulated *TLR4* gene expression through the NF-κB pathway [19]. This confirms the activation of the NF-κB pathway that has been demonstrated in HM-treated THP-1 cells [15]. Recently reported as the site of apoptosis induction in THP-1 cells through both intrinsic and extrinsic pathways [17] and to highlight the TLR4-dependent cell death mechanism underlying the HM anti-proliferative effect, apoptotic effects of HM in TLR4-positive THP-1 cells were studied and compared with TLR4-negative HEK293 cells. We observed that at the highest concentration (500 μg/mL) used, HM induced an apoptotic status based on Annexin V/PI double staining in THP-1 cells, which presented a higher HM-treated cell population in the late apoptotic phase than in the early apoptotic phase. In contrast to THP-1 cells, HM did not induce apoptosis in HEK293 cells, even when used at the highest concentration, providing evidence that HM-induced apoptosis occurred in a TLR4-dependent manner. Compared with the untreated cell population, no increase in necrotic cells was detected in the HM-treated THP-1 cells, indicating no cell damage-related conditions. It has been widely reported that early apoptotic cells retain membrane integrity, while late apoptotic cells present compromised membranes [43]. Recently, many natural herbal products have been found to induce apoptosis via the intrinsic and/or extrinsic pathways [36, 44]. With regards to activation of the extrinsic apoptotic pathway, we showed that the highest HM concentration induced the cleavage of caspase-8 in THP-1 cells, resulting in the detection of three cleaved-caspase 8 fragments of 18, 41, and 43 kDa in size. A previous study also reported that caspase-8 was cleaved in THP-1 cells treated with a low HM concentration; however, no cleavage of caspase-3 was detected [15]. The lack of cleavage of caspase-3 was associated with the low HM concentration used. The absence of induction of apoptosis in these low HM concentration-treated THP-1 cells was also observed in the present study. With regards to activation of the intrinsic apoptotic pathway, we showed that the highest HM concentration increased mitochondrial outer membrane permeability, which was indicated by evaluating mitochondrial transition pore opening activity. Activation of the HM-triggered intrinsic apoptotic pathway was confirmed by the cleavage of caspase-9, revealed by the concomitant detection of cleaved caspase-9 fragments of 35 and 47 kDa in size. Other studies have demonstrated that the apoptotic effects of neuromelanin (melanin originating from the brain), mainly occur through the intrinsic mitochondria-based pathway [45].

## Conclusions

In support of previous studies testing other melanins as anti-cancer agents [3, 40], our present findings indicate that HM from *Nigella sativa L*, may be considered as a drug candidate for the development of a safe natural anti-proliferative anti-cancer agent. The anti-proliferative effects of HM occur through cell cycle interference in a TLR4-independent manner without damaging the cells, reducing the risk of inflammation and other side effects. Since the pro-apoptotic effects of HM are observed only at non-physiological (high) concentrations, further studies including a detailed chemical and biological analysis to identify the effective functional group(s) within the molecular-component of HM may be undertaken to strengthen the observed anti-cancer action of HM.

## Data Availability

Data can be made available. Please contact the corresponding author.

## Abbreviations

ATP: Adenosine triphosphate
CDK: Cyclin-dependent kinase
DMEM: Dulbecco’s modified Eagle medium
EDTA: Ethylenediaminetetraacetic acid
FACS: Fluorescence-activated cell sorting
FBS: Fetal bovine serum
FITC: Fluorescein isothiocyanate
HEK: Human embryonic kidney
HM: Herbal melanin
IL: Interleukin
LPS: Lipopolysaccharides
PBS: Phosphate-buffered saline
PE: Phycoerythrin
PI: Propidium iodide
PMSF: Phenylmethylsulfonyl fluoride
RPMI: Roswell Park Memorial Institute
SDS-PAGE: Sodium dodecyl sulfate-polyacrylamide gel electrophoresis
TLR: Toll-like receptor
